# Association between COVID‐19 and consistent mask wearing during contact with others outside the household—A nested case–control analysis, November 2020–October 2021

**DOI:** 10.1111/irv.13080

**Published:** 2023-01-05

**Authors:** Ashley H. Tjaden, Sharon L. Edelstein, Naheed Ahmed, Lydia Calamari, Keerti L. Dantuluri, Michael Gibbs, Amy Hinkelman, Morgana Mongraw‐Chaffin, John W. Sanders, Sharon Saydah, Ian D. Plumb, the COVID‐19 Community Research Partnership Study Group

**Affiliations:** ^1^ Milken Institute School of Public Health, Biostatistics Center George Washington University Rockville Maryland USA; ^2^ Department of Population Health NYU Grossman School of Medicine New York City New York USA; ^3^ Carolinas Medical Center Atrium Health Charlotte North Carolina USA; ^4^ Department of Pediatrics (Infectious Diseases) Levine Children's Hospital, Atrium Health Charlotte North Carolina USA; ^5^ Campbell University School of Osteopathic Medicine Lillington North Carolina USA; ^6^ Department of Epidemiology and Prevention Wake Forest School of Medicine Winston‐Salem North Carolina USA; ^7^ Section on Cardiovascular Medicine, Department of Medicine Wake Forest University School of Medicine Winston‐Salem North Carolina USA; ^8^ U.S. Centers for Disease Control and Prevention COVID‐19 Response Atlanta Georgia USA

**Keywords:** COVID‐19, face masks, non‐pharmaceutical interventions, SARS‐CoV‐2, vaccination

## Abstract

**Background:**

Face masks have been recommended to reduce SARS‐CoV‐2 transmission. However, evidence of the individual benefit of face masks remains limited, including by vaccination status.

**Methods:**

As part of the COVID‐19 Community Research Partnership cohort study, we performed a nested case–control analysis to assess the association between self‐reported consistent mask use during contact with others outside the household and subsequent odds of symptomatic SARS‐CoV‐2 infection (COVID‐19) during November 2020–October 2021. Using conditional logistic regression, we compared 359 case‐participants to 3544 control‐participants who were matched by date, adjusting for enrollment site, age group, sex, race/ethnicity, urban/rural county classification, and healthcare worker occupation.

**Results:**

COVID‐19 was associated with not consistently wearing a mask (adjusted odds ratio [aOR] 1.49; 95% confidence interval [CI] [1.14, 1.95]). Compared with persons ≥14 days after mRNA vaccination who also reported always wearing a mask, COVID‐19 was associated with being unvaccinated (aOR 5.94; 95% CI [3.04, 11.62]), not wearing a mask (aOR 1.62; 95% CI [1.07, 2.47]), or both unvaccinated and not wearing a mask (aOR 9.07; 95% CI [4.81, 17.09]).

**Conclusions:**

Our findings indicate that consistent mask wearing can complement vaccination to reduce the risk of COVID‐19.

## 1 INTRODUCTION

Non‐pharmaceutical interventions (NPIs), including wearing face masks, have been recommended to decrease the risk of exposure and subsequently reduce SARS‐CoV‐2 transmission during the COVID‐19 pandemic.^1^, ^2^, ^3^, ^4^, ^5^ Although COVID‐19 vaccines are effective in preventing severe disease,^6^, ^7^ individual protection against infection wanes over time and is lower against new Omicron variants.^6^ Face masks are a valuable tool in reducing the risk of exposure to SARS‐CoV‐2 both by blocking emission of respiratory particles from a person with COVID‐19 and by providing individual protection to the wearer against exposure from others.^5^, ^8^, ^9^, ^10^

Evidence for the benefit of face masks includes ecologic analyses of the impact of mask policies at national,^11^ state,^12^ and county levels,^13^ in schools,^14^, ^15^, ^16^ estimates of cost‐saving,^9^, ^17^ models estimating the effect of masks in attenuating SARS‐CoV‐2 reproductive number,^8^, ^18^, ^19^ and a cluster randomized trial of a community‐based intervention.^20^ Several observational studies have also indicated an individual benefit in protecting the wearer.^1^, ^3^, ^21^, ^22^, ^23^ However, these studies have generally used retrospective questions concerning previous behavior, and there is limited information on the combined benefit of masks and vaccination.

Using real‐world data from the COVID‐19 Community Research Partnership (CRP), a multi‐site, prospective cohort study, we assessed the association between self‐reported consistent mask use when interacting with others outside the household and the odds of COVID‐19 during November 2020–October 2021. We assessed whether the magnitude of this effect differed by whether participants reported a known contact with a person with SARS‐CoV‐2 and by participant vaccination status. Lastly, we assessed how mask‐wearing behavior changed before and after a self‐reported infection as well as the proportion of individuals who reported they had SARS‐CoV‐2 exposure prior to testing positive.

## 2 METHODS

### 2.1 Study participants

Participants were enrolled in the COVID‐19 CRP, a multi‐site prospective cohort study based in the mid‐Atlantic and southern United States.^24^ Adults affiliated with 10 participating healthcare systems were invited to enroll in the study beginning in April 2020, including patients within health systems; some other community members also enrolled in the study after local advertising via the healthcare system website. Participants completed a daily electronic survey and gave permission to access their electronic health record (EHR) data. A subgroup of adult participants aged ≥18 years were invited to provide monthly dried blood spot (DBS) specimens for serology testing at the following six healthcare systems: Atrium Health (North Carolina), MedStar Health (Maryland), University of Maryland (Maryland), Tulane University (Louisiana), University of Mississippi Medical Center (Mississippi), and Wake Forest Baptist Health (North Carolina).

### 2.2 Data collection

Study data were collected and maintained via a secure, HIPAA‐compliant, online platform, and all participants provided informed consent. The study was reviewed and approved by the Wake Forest Institutional Review Board (IRB), which served as the central IRB for this study (see 45 C.F.R. Part 46; 21 C.F.R. Part 56). The study is registered with ClinicalTrials.gov (NCT04342884).

At enrollment, participants reported age, sex, race, ethnicity, educational level, healthcare worker status, and county of residence. We classified counties of residence as urban, rural, or suburban based on population density estimates. Underlying health conditions were assessed from a combination of electronic surveys and EHR data (Table S1). Daily electronic surveys included questions on the presence of any COVID‐19‐like symptoms, any new test results for COVID‐19, close contact with a person with COVID‐19, and consistent mask use when in contact with persons outside the household (Box S1). We refer to “index date” as the symptom onset date preceding a self‐reported positive SARS‐CoV‐2 virologic test for case‐participants and the corresponding matched survey entry date for control‐participants.

Vaccination status was ascertained using a combination of data from the daily survey and a supplemental survey distributed in September 2021, and included date, dose, and specific COVID‐19 vaccine product received. For a subset of participants with vaccination information available in the EHR, EHR information was used instead if vaccine doses were reported in the EHR that were not reported in the survey. We categorized vaccination status on the index date as “vaccinated” if ≥14 days had elapsed since the second mRNA dose, unvaccinated if no COVID‐19 vaccine was received, or “other” if only one dose or an alternative product was received, or if vaccination status was undetermined (e.g., conflicting or inadequate information provided for categorization). We defined “vaccinated” based on receipt of mRNA vaccines for analysis purposes because these vaccines had higher estimated effectiveness during the study period^25^ and because <5% of participants within our study population received non‐mRNA COVID‐19 vaccine products. Participants were considered to be unvaccinated if they reported no vaccination before the index date and if there was no evidence of receipt of a COVID‐19 vaccine from any of the three available sources.

Serology was evaluated using DBS specimens collected at home by finger prick on a monthly basis using Whatman 5‐spot DBS cards. All viable specimens were evaluated for anti‐spike antibody using a EUROIMMUN qualitative assay for SARS‐CoV‐2 anti‐spike immunoglobulin G (IgG); any DBS card with a positive result underwent reflex testing of a different DBS on the same card for anti‐nucleocapsid antibody using a qualitative Roche pan‐Ig assay.^26^ Both serologic assays used were internally validated for use with DBS cards; evaluation of DBS using the EUROIMMUN assay is reported elsewhere.^27^, ^28^

### 2.3 Analysis design

To compare characteristics and behaviors associated with SARS‐CoV‐2 infection, we performed a nested case–control analysis within the study cohort. We opted for a nested case–control approach to limit potential biases from differences in the risk of infection over time, different follow‐up periods by infection status, variable survey completion, and differences in completion of serology tests. By conditioning on the availability of serology results, this approach was also designed to limit potential selection bias related to the use and return of serology kits by study participants.^29^

Participants were eligible for inclusion in the analysis if all the following criteria were met: (1) age ≥ 18 years; (2) returned at least two serology test kits; (3) the first SARS‐CoV‐2 serology result was negative for anti‐spike antibody (indicating no evidence of infection or vaccination) or positive for anti‐spike antibody but negative for anti‐nucleocapsid antibody (indicating previous COVID‐19 vaccination but no prior evidence of infection); and (4) there was no evidence of previous SARS‐CoV‐2 infection from the daily survey or EHR (diagnosis or positive lab result in EHR) on or before the day of the first available study serology test result.

Case‐participants were eligible if they self‐reported a positive COVID‐19 test (indicating a positive SARS‐CoV‐2 antigen or nucleic acid amplification test result) ≥14 days after an initial serology result that did not indicate infection, and if any new COVID‐19‐like symptoms were reported. We defined COVID‐19‐like symptoms as fever, chills, cough, shortness of breath, fatigue, muscle pain, headache, loss of taste/smell, sore throat, congestion/runny nose, nausea/vomiting, or diarrhea. To account for a baseline level of symptoms in some participants, we defined new COVID‐19‐like symptoms as any symptom reported for the first time in a 7‐day period if the symptom onset was during the 10 days before the self‐reported positive test date. This definition was based on review of the timing of symptoms relative to the reported test date (Figure S1). For analytic purposes, we considered the estimated date of SARS‐CoV‐2 infection for case‐participants to be the symptom onset date.

To account for differences in the risk of infection over the 12‐month study period, we matched each case‐participant with up to 10 control‐participants who had an eligible survey entry on the same date as the case‐participant's date of symptom onset. Control‐participants were eligible if they had no evidence of having a SARS‐CoV‐2 infection (never self‐reported a positive test, no serologic evidence of SARS‐CoV‐2 infection, no EHR diagnosis, and no positive lab result in EHR). Additionally, control‐participants were eligible to be matched on a particular date if they had a survey entry on that date that was followed by a negative serology result in the subsequent 30–90 days, and if there was ≥1 other survey entry during the previous 10 days. We used an optimal matching algorithm, without replacement, maximizing the number of case‐participants included.^30^

We defined consistent mask use as responding “yes” to the question, “In the last 24 hours, have you worn a face mask or face covering every time you interacted with others (not in your household) within a distance of less than 6 feet?”. We included consistent mask use as an exposure variable if reported during the 10 days before the match date (index date); as a secondary analysis, we also reported consistent mask use during the 10 days after the index date (Box S1).

We defined known a COVID‐19 exposure as a participant responding “yes” to the question, “Did you have close contact with someone who has tested positive for COVID‐19 infection?”, during the 10 days before the index date. We determined the likely timing of the reported contact according to the date of the report and how long ago the known exposure was reported to have occurred (last 24 h, last 7 days, 1–2 weeks ago, or >2 weeks ago) (Box S1). For participants who did not self‐identify as healthcare workers and who reported a known close contact with a person with COVID‐19, participants were asked whether the proximity was <6 feet, and whether the duration of exposure was ≥15 minutes.

### 2.4 Statistical analysis

After summarizing mask use over time among case‐ and control‐participants, we used conditional logistic regression to compare characteristics of case‐ and control‐participants while accounting for matching by date. In multivariable models comparing characteristics among case‐ and control‐participants, we adjusted for all other characteristics of interest. In multivariable models of consistent mask use or known COVID‐19 contact, we adjusted for characteristics that either differed between case‐ and control‐participants or were hypothesized to lead to differential risk of SARS‐CoV‐2 infection: enrollment site, age group, sex, race/ethnicity, healthcare worker occupation, rurality of county (rural, suburban, or urban), and vaccination status on the index date. We used conditional logistic regression models with interaction terms to assess whether odds ratios (OR) for consistent mask use varied by reported COVID‐19 exposure, vaccination status, or healthcare worker occupation. We subsequently stratified analyses by these groups and estimated odds of SARS‐CoV‐2 infection. Because of sparse data, we used unconditional logistic regression to assess the associations between COVID‐19 and the proximity and duration of known COVID‐19 exposure among non‐healthcare workers, we used unconditional logistiadjusted by 3‐month quarter of index date, age group, sex, race/ethnicity, county classification, and vaccination status.

In addition, we repeated the main analyses evaluating the association between known exposure with COVID‐19 and consistent mask use, restricted to participants who were not healthcare workers. To address robustness of our findings under an alternative case definition, we performed a sensitivity analysis of the main estimates limited to case‐participants who also had serologic evidence of new SARS‐CoV‐2 infection (indicated by positive anti‐nucleocapsid antibody) during the 30–90 days following the date of symptom onset associated with the self‐reported positive SARS‐CoV‐2 virologic test.

All statistical analyses were performed using R Statistical Software (Version 4.0.3; R Foundation for Statistical Computing, Vienna, Austria).

## 3 RESULTS

### 3.1 Study participants

**FIGURE 1:**
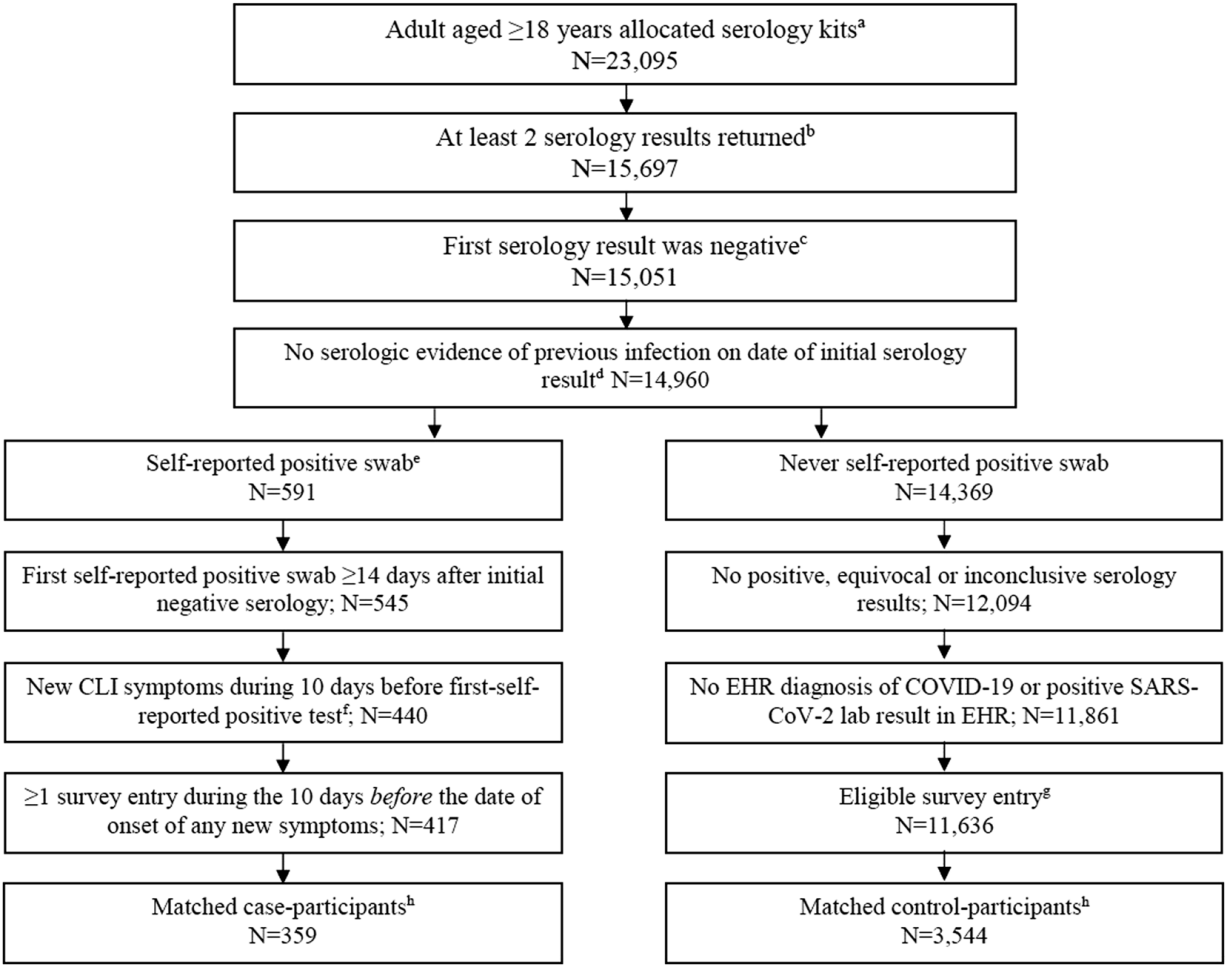
Participants included in the analysis. CLI, COVID‐19‐like illness; EHR, electronic health record. ^a^Of the 30,372 participants enrolled in the CDC study, 28,026 participants did not report participating in a vaccine clinical trial nor self‐report a previous COVID‐19 diagnosis at the time of enrollment. Of these, 23,095 were allocated at least one serology test kit. ^b^See Table S2 for comparison of participants. ^c^Defined as negative anti‐S, or positive anti‐S and negative anti‐N. ^d^Self‐reported through daily survey or EHR diagnosis or lab results. ^e^See Figure S1. ^f^Defined as any symptom in daily survey not reported during the previous 7 days (see Table S3). ^g^Defined as daily survey entry with ≥1 other entry during previous 10 days and ≥1 negative serology result during the next 30–90 days. ^h^Matched on date of survey (1 case to up to 10 controls)

Among 23,095 participants who were allocated serology test kits, 15,697 (68.0%) had at least two serology test results. After applying exclusion criteria, 359 case‐participants were matched to 3,544 control‐participants (Figure 1). The median number of controls per case was 10 (range 2–10).

Compared with control‐participants, in an adjusted model, case‐participants were more likely to be younger, reside in a county classified as rural, and less likely to have received a second mRNA vaccine dose ≥14 days earlier (Table 1). There were no differences in case‐ compared with control‐participants by sex, healthcare worker occupation, or presence of an underlying health condition. In a multivariable model, symptomatic SARS‐CoV‐2 infection was associated with younger age, living in a rural or suburban county, health system, and being unvaccinated (Table 1).

**TABLE 1:** Overall characteristics of case‐participants and control‐participants

	Case‐participants^a^ (N = 359)	Control‐participants^b^ (N = 3544)	Univariable conditional OR [95% CI]	Multivariable conditional OR [95% CI]^c^
Age group in years				
18–39	109 (30.4%)	736 (20.8%)	Ref	Ref
40–54	111 (30.9%)	820 (23.1%)	0.91 [0.68, 1.20]	0.73 [0.49, 1.09]
55–64	77 (21.4%)	705 (19.9%)	**0.72 [0.52, 0.98]**	**0.60 [0.38, 0.93]**
≥65	62 (17.3%)	1283 (36.2%)	**0.32 [0.23, 0.44]**	**0.38 [0.24, 0.61]**
Sex				
Female	221 (61.6%)	2215 (62.5%)	Ref	Ref
Male	138 (38.4%)	1329 (37.5%)	1.04 [0.83, 1.30]	1.14 [0.84, 1.56]
Race and ethnicity				
White, non‐Hispanic	297 (82.7%)	2970 (83.8%)	Ref	Ref
Black, non‐Hispanic	17 (4.7%)	263 (7.4%)	0.65 [0.40, 1.08]	0.78 [0.41, 1.49]
Hispanic	26 (7.2%)	136 (3.8%)	**1.94 [1.24, 3.02]**	1.32 [0.66, 2.64]
Other^d^	19 (5.3%)	175 (4.9%)	1.09 [0.67, 1.78]	1.08 [0.55, 2.13]
County of residence^e^				
Urban	140 (39.0%)	1868 (52.7%)	Ref	Ref
Suburban	104 (29.0%)	901 (25.4%)	**1.56 [1.19, 2.04]**	**1.69 [1.17, 2.45]**
Rural	115 (32.0%)	775 (21.9%)	**2.02 [1.55, 2.63]**	**1.54 [1.05, 2.27]**
Household size^f^				
1–2	108 (42.2%)	1474 (55.2%)	Ref	Ref
>2	148 (57.8%)	1197 (44.8%)	**1.74 [1.33, 2.27]**	1.26 [0.91, 1.75]
Highest educational level^f^				
No college degree	105 (30.0%)	681 (19.8%)	Ref	Ref
College degree	245 (70.0%)	2758 (80.2%)	**0.57 [0.44, 0.72]**	0.87 [0.61, 1.24]
Underlying health condition^g^				
No	87 (24.4%)	842 (23.9%)	Ref	Ref
Yes	270 (75.6%)	2688 (76.1%)	0.97 [0.75, 1.25]	0.79 [0.56, 1.13]
Healthcare worker^h^				
No	284 (79.1%)	2926 (82.6%)	Ref	Ref
Yes	75 (20.9%)	618 (17.4%)	1.25 [0.96, 1.64]	1.01 [0.68, 1.49]
Vaccination status^i^				
≥14 days after 2nd mRNA dose	158 (44.0%)	1959 (55.3%)	Ref	Ref
No COVID‐19 vaccination	161 (44.8%)	1178 (33.2%)	**8.66 [5.55, 13.51]**	**6.59 [3.56, 12.20]**
Other	40 (11.1%)	407 (11.5%)	**2.52 [1.68, 3.78]**	**1.96 [1.13, 3.41]**
Health system				
Atrium Health	67 (18.7%)	508 (14.3%)	Ref	Ref
University of Maryland	26 (7.2%)	606 (17.1%)	**0.23 [0.14, 0.38]**	**0.24 [0.14, 0.44]**
MedStar Health	62 (17.3%)	1333 (37.6%)	**0.27 [0.19, 0.39]**	**0.33 [0.21, 0.53]**
University of Mississippi/Tulane University	8 (2.2%)	37 (1.0%)	1.25 [0.56, 2.81]	1.38 [0.53, 3.62]
Wake Forest Health	196 (54.6%)	1060 (29.9%)	**1.58 [1.16, 2.15]**	**1.70 [1.18, 2.58]**
Month of index date^j^				
November, 2020	6 (1.7%)	60 (1.7%)	‐	‐
December, 2020	48 (13.4%)	480 (13.5%)	‐	‐
January, 2021	53 (14.8%)	530 (15.0%)	‐	‐
February, 2021	20 (5.6%)	200 (5.6%)	‐	‐
March, 2021	18 (5.0%)	180 (5.1%)	‐	‐
April, 2021	15 (4.2%)	150 (4.2%)	‐	‐
May, 2021	5 (1.4%)	50 (1.4%)	‐	‐
June, 2021	3 (0.8%)	30 (0.8%)	‐	‐
July, 2021	35 (9.7%)	350 (9.9%)	‐	‐
August, 2021	112 (31.2%)	1120 (31.6%)	‐	‐
September, 2021	44 (12.3%)	394 (11.1%)	‐	‐
Response rate^k^	0.82 ± 0.24	0.81 ± 0.25	‐	‐

Abbreviations: CI, confidence interval; OR, odds ratio; Ref, Reference.

^a^ Case‐participants self‐reported a positive viral test (indicating a positive SARS‐CoV‐2 antigen or nucleic acid amplification test result) and new COVID‐19‐like symptoms.

^b^ Control‐participants had no serologic or virologic evidence of SARS‐CoV‐2 infection, and were matched to case‐participants by survey date.

^c^ Adjusted for all other variables in the table (with the exception of month of index date and survey response rate).

^d^ Other race/ethnicity includes American Indian or Alaskan Native, Asian or Pacific Islander, mixed race/ethnicity, and participants who chose not to specify their race/ethnicity.

^e^ Defined using population density estimates for county of residence.

^f^ Educational level and household size were only available for a subset of participants who completed the supplemental survey including this information.

^g^ Defined as any comorbidity that was self‐reported or in electronic health records (autoimmune disease [N = 838], cancer [N = 648], cardiovascular disease [N = 1612], diabetes [N = 456], immunocompromised [N = 90], liver disease [N = 144], renal disease [N = 180], obesity [N = 1167], pulmonary disease [N = 823], neurologic disease [N = 610], substance use disorder [N = 178], mental health condition [N = 369], and other underlying health conditions associated with elevated risk of COVID‐19 [N = 22]).

^h^ Self‐reported at enrollment.

^i^ Vaccination status on the match/index date. 'Other' includes non‐mRNA vaccine products, receipt of only one COVID‐19 vaccine dose, or undetermined vaccination status.

^j^ Index date is the match date for cases (date of symptom onset) and controls (corresponding daily survey entry).

^k^ Daily syndromic survey response rate for the 10 days preceding the index date.

### 3.2 Association between reported exposure and COVID‐19

**FIGURE 2:**
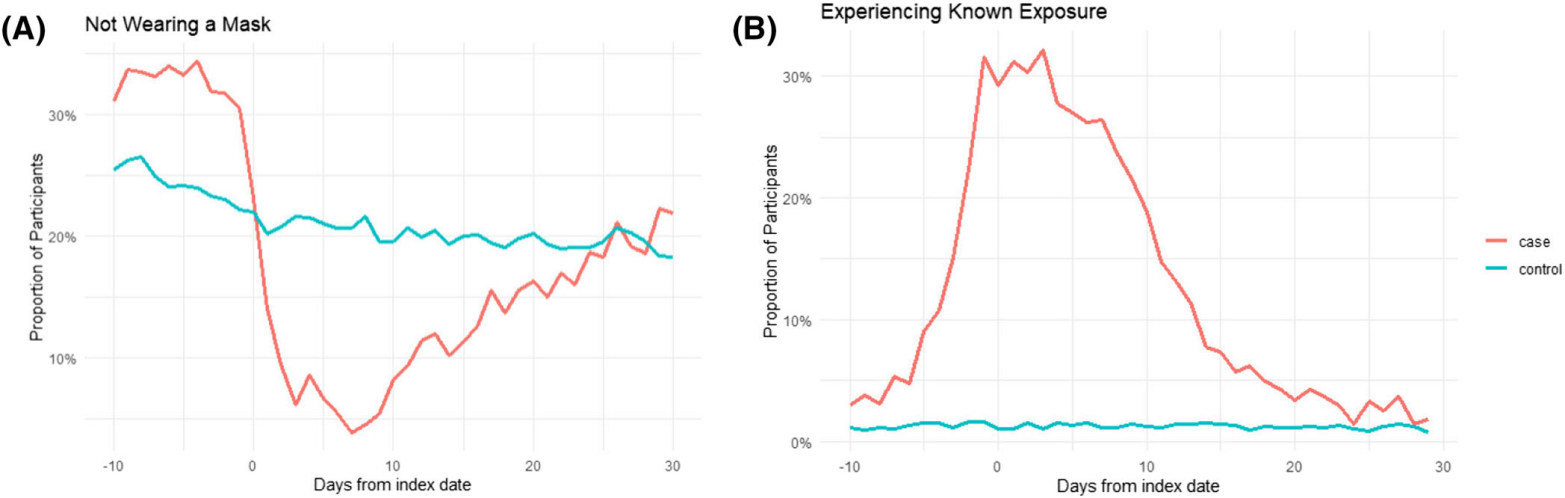
(A) Mask use and (B) reported exposure before and after symptom onset that preceded a self‐reported SARS‐CoV‐2‐positive swab, compared with control‐participants with negative SARS‐CoV‐2 serology

The daily survey response rate in the 10 days preceding the index date was similar for case‐participants (82%) and control‐participants (81%). Among case‐participants, 40% (143/359) reported a known exposure to a person with COVID‐19 during this period compared with 4% (142/3544) of control‐participants (Table 2). The proportion of case‐participants reporting a known exposure began to increase 5 days preceding symptom onset and was highest in the day preceding and the first few days following new symptom onset (Figure 2B).

**TABLE 2:** Associations between COVID‐19 and mask use or known COVID‐19 contact during the 10 days before the index date, November 2020–October 2021

	Case‐participants (N = 359)^a^	Control‐participants (N = 3544)^b^	Unadjusted cOR [95% CI]^c^	Adjusted cOR [95% CI]^d^
Contact with others outside household without a mask^e^				
No	148 (41.2%)	1764 (49.8%)	Ref	Ref
Yes	211 (58.8%)	1780 (50.2%)	**1.57 [1.22, 2.03]**	**1.49 [1.14, 1.95]**
Timing of contact with others outside household without a mask^f^				
6–10 days before index date				
No	171 (49.3%)	1882 (56.7%)	Ref	Ref
Yes	176 (50.7%)	1435 (43.3%)	**1.58 [1.23, 2.05]**	**1.44 [1.09, 1.89]**
1–5 days before index date				
No	174 (49.7%)	2073 (59.2%)	Ref	Ref
Yes	176 (50.3%)	1431 (40.8%)	**1.65 [1.28, 2.12]**	**1.52 [1.16, 2.00]**
By vaccination status and mask use^g^				
Vaccinated, masked	37 (11.6%)	630 (20.1%)	Ref	Ref
Vaccinated, not masked	121 (37.9%)	1329 (42.4%)	1.41 [0.95, 2.09]	**1.62 [1.07, 2.47]**
Unvaccinated, masked	90 (28.2%)	875 (27.9%)	**8.32 [4.46, 15.50]**	**5.94 [3.04, 11.62]**
Unvaccinated, not masked	71 (22.3%)	303 (9.7%)	**14.39 [8.01, 25.85]**	**9.07 [4.81, 17.09]**
Known exposure to a person with COVID‐19?^h^				
No	216 (60.2%)	3402 (96.0%)	Ref	Ref
Yes	143 (39.8%)	142 (4.0%)	**17.95 [13.23, 24.36]**	**17.55 [12.31, 25.03]**
Timing of known exposure to a person with COVID‐19				
6–10 days before index date				
No	315 (91.0%)	3218 (97.4%)	Ref	Ref
Yes	31 (9.0%)	85 (2.6%)	**3.79 [2.44, 5.90]**	**2.86 [1.72, 4.74]**
1–5 days before index date				
No	221 (62.1%)	3389 (97.3%)	Ref	Ref
Yes	135 (37.9%)	95 (2.7%)	**21.00 [14.73, 29.94]**	**21.31 [14.57, 31.18]**

Abbreviations: CI, confidence interval; cOR, conditional odds ratio; Ref, Reference.

^a^ Case‐participants self‐reported a positive viral test (indicating a positive SARS‐CoV‐2 antigen or nucleic acid amplification test result) and new COVID‐19‐like symptoms.

^b^ Control‐participants had no serologic or virologic evidence of SARS‐CoV‐2 infection, and were matched to case‐participants by survey date.

^c^ Conditional logistic regression. Each category represents a separate model.

^d^ Adjusted for enrollment site, age group, sex, race/ethnicity, urban/rural county classification, healthcare worker occupation, and vaccination status.

^e^ Report of one or more interactions outside household without wearing a mask in the 1–10 days preceding the index date. Among participants who reported some contact with others outside the household, the adjusted odds ratio for infection if not wearing a mask was 1.44 (95% CI [1.10, 1.89]).

^f^ Excludes participants with no survey responses during the specified time period.

^g^ Vaccination status at the time of match. Vaccinated: ≥14 days after second dose of mRNA vaccine; Unvaccinated: no doses of COVID‐19 vaccine reported. Excludes participants who were partially vaccinated or received a different product.

^h^ Report of one or more known exposure to a person with COVID‐19 in the 1–10 days preceding the index date.

Reported exposure to a person with COVID‐19 was strongly associated with greater odds of symptomatic SARS‐CoV‐2 infection compared with those with no reported exposure (adjusted odds ratio [aOR] 17.55; 95% confidence interval [CI] [12.31, 25.03]) (Table 2). The association was stronger for those who reported a known exposure during 1–5 days (aOR 21.31; 95% CI [14.57, 31.18]) compared with known exposures reported in the 6–10 days preceding the index date (aOR 2.86; 95% CI [1.72, 4.74]). Among non‐healthcare workers who were asked follow‐up questions about their known exposure, duration of contact ≥ 15 minutes, proximity of contact ≤ 6 feet, and contact outside the workplace were all associated with increased odds of SARS‐CoV‐2 infection (Table S1). Among non‐healthcare workers with a known exposure, duration of contact ≥ 15 minutes was associated with 3.5‐fold greater odds of infection compared with those with a shorter duration of exposure (aOR 3.50; 95% CI [1.70, 7.41]). Contact outside one's workplace was associated with greater odds of infection (aOR 3.95; 95% CI [1.70, 9.59]).

### 3.3 Association between reported mask wearing and COVID‐19

During the 10 days preceding the index date, 59% (211/359) of case‐participants and 50% (1780/3544) of control‐participants reported not wearing a mask during one or more interactions with others outside the household (Table 2). On the date of the match, the proportion of case‐participants who reported not wearing a mask sharply declined; after the date of the match, fewer case‐participants than control‐participants reported days not wearing a mask (Figure 2A). Whereas mask use remained constant over time for controls, the proportion of case‐participants who reported not wearing a mask on any day decreased from 59% in the 10 days preceding the index date to 21% (75/351) during the 1–5 days following the index date, and to 12% (45/351) in the 6–10 days following the index date.

After adjustment, not wearing a mask during one or more interactions with others outside the household was associated with increased odds of SARS‐CoV‐2 infection (aOR 1.49; 95% CI [1.14, 1.95]). The association was similar for any days reported without wearing a mask during 1–5 or 6–10 days preceding the index date. Similarly, after excluding participants who did not report any contact with others outside the household 1–10 days preceding the index date, not consistently wearing a mask was associated with higher odds of SARS‐CoV‐2 infection (aOR 1.44; 95% CI [1.10, 1.89]) (Table 2, footnote).

The associations between COVID‐19 and not consistently wearing a mask were similar among those with or without reported close contact with a person with COVID‐19 (aOR 1.53; 95% CI [1.10, 2.15] vs. OR 1.41; 95% CI [0.73, 2.76], respectively) and appeared stronger among non‐healthcare workers compared with healthcare workers (aOR 1.66; 95% CI [1.23, 2.24] vs. OR 1.19; 95% CI [0.66, 2.16], respectively). Among unvaccinated participants, COVID‐19 was associated with not consistently wearing a mask during contact outside the household (aOR 1.81; 95% CI [1.21, 2.70]); among participants who were ≥14 days after second mRNA vaccine dose, the association between COVID‐19 and not wearing a mask had weaker statistical evidence (aOR 1.49; 95% CI [0.99, 2.28]). Despite some differences in ORs between subgroups, associations between COVID‐19 and not consistently wearing a mask did not differ statistically by whether there was known exposure, healthcare worker occupation, or by vaccination status (Table 3, footnotes).

**TABLE 3:** Associations between COVID‐19 and mask use or known COVID‐19 contact during the 10 days before the index date^a^, November 2020–October 2021, by subgroup

Subgroup	Case‐participants^b^ (N = 359)	Control‐participants^c^ (N = 3544)	Unadjusted OR^d^ [95% CI]	Adjusted OR^e^ [95% CI]
**All participants**				
*By known exposure* ^f^				
No close contact reported	216 (60.2%)	3402 (96.0%)	**1.60 [1.16, 2.21]**	**1.53 [1.10, 2.15]**
Close contact reported	143 (39.8%)	142 (4.0%)	**1.82 [1.07, 3.12]**	1.41 [0.73, 2.76]
*By vaccination status* ^g^				
Unvaccinated	161 (50.5%)	1178 (37.6%)	**1.78 [1.23, 2.54]**	**1.81 [1.21, 2.70]**
≥14 days after 2nd mRNA dose	158 (49.5%)	1959 (62.4%)	1.40 [0.95, 2.10]	1.49 [0.99, 2.28]
*By healthcare worker status* ^h^				
Healthcare worker	75 (20.9%)	618 (17.4%)	1.25 [0.73, 2.18]	1.19 [0.66, 2.16]
Non‐healthcare worker	284 (79.1%)	2926 (82.6%)	**1.71 [1.28, 2.27]**	**1.65 [1.23, 2.24]**
**Non‐healthcare workers** ^h^				
*By known exposure* ^f^				
No close contact reported	177 (62.3%)	2857 (97.6%)	**1.81 [1.27, 2.59]**	**1.76 [1.22, 2.56]**
Close contact reported	107 (37.7%)	69 (2.4%)	1.30 [0.63, 2.68]	1.22 [0.48, 3.10]
*By vaccination status* ^g^				
Unvaccinated	139 (56.3%)	1043 (40.0%)	**1.92 [1.30, 2.81]**	**2.05 [1.34, 3.13]**
≥14 days after 2nd mRNA dose	108 (43.7%)	1567 (60.0%)	1.62 [1.00, 2.75]	1.57 [0.95, 2.71]

Abbreviations: CI, confidence interval; OR, odds ratio.

^a^ Date of symptom onset for cases and date of corresponding syndromic survey entry for controls.

^b^ Case‐participants self‐reported a positive viral test (indicating a positive SARS‐CoV‐2 antigen or nucleic acid amplification test result) and new COVID‐19‐like symptoms.

^c^ Control‐participants had no serologic or virologic evidence of SARS‐CoV‐2 infection and were matched to case‐participants by survey date.

^d^ Odds ratios from stratified unconditional logistic regression models adjusted for month of index date. The reference group for all odds ratios was the group within the specified subgroup who reported always wearing a mask.

^e^ Adjusted for enrollment site, age group, sex, race/ethnicity, county classification, healthcare worker occupation, and vaccination status (except when used as the stratification variable).

^f^ Stratified by reporting a known exposure to COVID‐19 in the 10 days preceding the index date. The interaction term for no mask * known exposure (adjusted conditional logistic odds ratio) was 1.11 (95% CI [0.62, 1.97]).

^g^ Vaccination status at the time of match. Vaccinated: >14 days after second dose of mRNA vaccine; Unvaccinated: no doses of COVID‐19 vaccine reported. Excludes participants who were partially vaccinated or received a different product. Interaction term for no mask * vaccination status(adjusted conditional logistic odds ratio) was 1.03 (95% CI [0.52, 2.05]).

^h^ Healthcare worker occupation self‐reported at enrollment. Interaction term for no mask * healthcare worker occupation (adjusted conditional logistic odds ratio) was 1.22 (95% CI [0.71, 2.11]).

When participants were grouped by vaccination status and masking behavior, we observed a stepped increase in the odds of infection with decreasing mitigation efforts. Compared with participants who were ≥14 days after a second mRNA vaccine dose and also reported consistent mask use, the OR for developing COVID‐19 was 1.62 (95% CI [1.07, 2.47]) if vaccinated without consistent mask use, 5.94 (95% CI [3.04, 11.62]) if consistently wearing a mask but unvaccinated, and 9.07 (95% CI [4.81, 17.09]) if unvaccinated and not consistently wearing a mask (Table 2).

### 3.4 Additional analyses

To assess representativeness within the study cohort, we compared characteristics of participants by whether they had at least two serology test results and were therefore potentially eligible. Overall characteristics of participants were similar by whether serology results were available. However, those with at least two serology test results were more likely to be non‐Hispanic White, compared with other participants (Table S2). Additional analyses to address potential limitations in reliance on self‐reported positive tests are summarized in Tables S3 and S4. Because participants were only asked to report a new test result since the most recent survey entry and were not directly asked about the collection date, a gap since the most recent survey could lead to an incorrect test date. However, >95% of case‐participants had a previous survey entry within the previous 2 days (Table S3). To address potential reliance on participants' report of a positive test, we performed a sensitivity analysis limited to case‐participants who also had serologic evidence of new infection. Among case‐participants, 77% (278/359) had an available serology result in the 30–90 days after symptom onset, of whom 74% (205/278) had serologic evidence of new infection. In an analysis comparing association with infection among this subset of case‐participants with their corresponding control‐participants, the aOR for COVID‐19 among participants not consistently wearing a mask was 1.86 (95% CI [1.29, 2.70]) overall, 1.87 (95% CI [1.21, 2.86]) among unvaccinated participants, and 2.62 (95% CI [1.21, 6.50]) among those ≥14 days after a second mRNA dose (Table S4).

## 4 DISCUSSION

In this prospective nested case–control analysis, not consistently wearing a mask during contact with others outside the household was associated with approximately 49% higher odds of COVID‐19 during November 2020–October 2021. We found that this association was similar whether or not there was reported close contact with a person with COVID‐19, and whether or not the participant was a healthcare worker. On its own, not consistently wearing a mask was also associated with COVID‐19 whether or not participants were vaccinated. Combined with vaccination status, a lack of consistent mask wearing was associated with a stepped increase in the odds of COVID‐19—participants who did not consistently wear a mask while unvaccinated had the highest odds of COVID‐19, whereas those consistently wearing a mask while vaccinated had the lowest odds of COVID‐19.

Overall, our findings are comparable with evidence from previous studies indicating that individual mask wearing is associated with decreased odds of infection.^3^, ^10^ However, few studies have used prospectively collected data^31^, ^32^ or evaluated mask use since the introduction of COVID‐19 vaccines or more recent variants.^1^, ^33^ Our finding of similar associations between not wearing a mask and COVID‐19 whether or not participants were vaccinated is consistent with findings from a case–control study conducted during 2021.^33^ Consistent with that study, we found that although the association between mask use and COVID‐19 was weaker among vaccinated participants, the modeled association did not differ significantly by vaccination status.

We found a clearer association between not consistently wearing a mask and COVID‐19 among vaccinated persons in a sensitivity analysis restricted to case‐participants with serologic evidence of infection. This might reflect masks providing additional protection against a subgroup of infections that elicit a more robust systemic immune response or that lead to more illness, because seroconversion is generally associated with more severe disease.^34^, ^35^ Although the study data were collected before predominance of the Omicron variant, we found evidence for the benefit of masks whether or not vaccine‐induced protection was present—completion of an mRNA primary series was highly protective against infection during the study period.^6^, ^7^ Our estimate of an approximately sixfold increased odds of COVID‐19 among unvaccinated participants is consistent with a previous analysis during the Delta‐predominant period.^36^

A majority of case‐participants were not able to identify known close contact with a person with COVID‐19, and we did not find a significant difference in odds of infection by whether previous contact was reported. Whereas mask effectiveness would be expected to vary greatly by the level of exposure,^37^ the similar odds associated with mask wearing by whether there was known or unknown contact indicate a protective role of masks even when contact with a case is unknown and also may suggest a limited awareness of COVID‐19 status of contacts. Among non‐healthcare workers who reported close contact with a person with COVID‐19, we found evidence that the odds of infection were highest when the exposure was within 6 feet or lasted at least 15 minutes. These associations are consistent with evidence that SARS‐CoV‐2 can be transmitted by infectious respiratory particles of varying sizes and predominantly by aerosols.^38^, ^39^ The increased risk associated with a COVID‐19 contact during the 5 days before symptom onset is consistent with estimates of the SARS‐CoV‐2 incubation period.^40^

Lack of consistent mask use during the 6‐10 days and during 1‐5 days before symptom onset  was associated with infection, suggesting that infection did not result from sudden changes in mask wearing. However, there was an abrupt decrease in contact with others without a mask following symptom onset, consistent with recommended precautions after SARS‐CoV‐2 infection.^41^ If COVID‐19 is confirmed, wearing a high‐quality mask is recommended during the 10 days after symptom onset when indoors around others at home and in public.^42^ We did not assess the benefit in protecting others by wearing a mask, but previous studies support this benefit, particularly when others are also wearing a mask.^5^ Although participants rapidly adopted mask use on the day of symptom onset, pre‐existing mask use would likely provide additional protection to others because transmission frequently occurs before symptoms develop.^43^ Mask use is recommended regardless of vaccination status following a known exposure and during high periods of transmission when exposures might be unknown.^42^, ^44^, ^45^

Our study has several strengths, including prospective collection of survey data to minimize recall bias, validation of a subset of testing results using serology, and a control group with serologic results indicating no evidence of SARS‐CoV‐2 infection. We also found that survey adherence was high and similar among case‐ and control‐participants in the days preceding the index date. Nevertheless, several internal limitations might have affected our findings. First, misclassification of cases might have occurred by relying on a self‐reported positive test. This might have weakened the association between mask use and infection, because this association became larger when restricted to a more robust definition of infection that also required serologic evidence. Second, imperfect sensitivity of the EUROIMMUN assay (estimated to be 91%^46^) might have led to misclassification of some control‐participants. To minimize potential misclassification of control‐participants, we excluded eligible control‐participants with inconclusive serologic results or evidence of SARS‐CoV‐2 infection in EHR data. Third, although prospective data collection is likely to have improved accuracy, we relied on self‐reported responses to identify exposures, behaviors, and vaccination status. A previous analysis of self‐reported vaccination data in this study indicated close agreement with vaccination information from the EHR.^47^ Fourth, our estimates might have been distorted by residual or unmeasured confounding. Similarity between adjusted and unadjusted estimates suggests that the effect of confounding was limited, and we adjusted for known COVID‐19 contact, which might act as a proxy for other protective behaviors. However, it remains plausible that the association between infection and mask use was affected by accompanying unmeasured behaviors that were also protective. Lastly, our analysis required assumptions concerning the timing of infection. We considered symptoms not reported in the previous 7 days to represent symptom onset and used symptom onset as a proxy for start of infection. Reported changes in symptoms and mask wearing over time support this analytic approach, but we were unable to verify these assumptions.

The scope of our analysis narrows potential conclusions from our findings. Our assessment of mask use was limited to reported contact with others outside the household without a mask at any time. This reflects an effort to minimize participant burden and encourage daily participation; the questions included in the daily survey therefore lacked granularity. For example, the survey question asked about mask use in “all” interactions outside the household and does not allow for reporting varying masking behaviors in different settings and does not include a “sometimes” response, which may more closely align with the behaviors of many participants. We also did not elicit the type of mask, and how well fitted it was, nor the frequency or duration of interactions. Previous studies have indicated that the greatest protection is offered by well‐fitting masks with high filtration, and use of other types of mask might have diluted our findings.^21^, ^48^ In assessing whether a mask‐wearing behavior was associated with decreased odds of infection if there was a known COVID‐19 contact, we were unable to distinguish whether the encounter with that infected contact was without a mask, or whether the person with COVID‐19 was a household member. We only assessed the individual risk of infection related to not wearing a mask and did not assess additional effects on transmission in the community. We were also unable to assess whether consistent mask wearing was associated with any other protective measures, although among cases there was no difference in reporting a known exposure between those who consistently reported wearing a mask and those who did not (Figure S2). Our assessment of attributes of known exposures was also limited to exposures that the participant considered to be “close contact.” Lastly, the generalizability of our findings might be limited because of geographic focus on east and southern regions of the United States, dependence on access to care for enrollment, overrepresentation of White non‐Hispanic and female participants, and by data collection before predominance of the Omicron variant.

Overall, we found that consistently wearing a mask when interacting with others outside the household was associated with lower odds of reported COVID‐19. Although the association between consistent mask use and COVID‐19 was similar regardless of vaccination status, vaccinated participants who reported consistent mask wearing had the lowest odds of infection, supporting a layered approach to prevention. When local community transmission is high, the Centers for Disease Control and Prevention recommend the use of well‐fitting masks or respirators indoors in public to limit the risk of exposure.^49^ Mask wearing to minimize the risk of exposure and vaccination to decrease susceptibility if exposed remain complementary strategies to reduce ongoing transmission of SARS‐CoV‐2.

## CONFLICTS OF INTEREST

The authors declare that they have no competing interests.

## ETHICS APPROVAL

The study was reviewed and approved by the Wake Forest Institutional Review Board (IRB), which served as the central IRB for this study (see U.S. federal regulations 45 C.F.R. Part 46; 21 C.F.R. Part 56). The study is registered with ClinicalTrials.gov (NCT04342884). All participants in the COVID‐19 Community Research Partnership provided written consent for participation. No identifying data from any individual person are contained in the manuscript.

## AUTHOR CONTRIBUTIONS

**Ashley H. Tjaden:** Conceptualization; formal analysis; methodology; writing‐original draft; writing‐review and editing. **Sharon L. Edelstein:** Conceptualization; formal analysis; methodology; writing‐original draft; writing‐review and editing. **Naheed Ahmed:** Methodology; writing‐review and editing. **Lydia Calamari:** Methodology; writing‐review and editing. **Keerti L. Dantuluri:** Methodology; writing‐review and editing. **Michael Gibbs:** Methodology; writing‐review and editing. **Amy Hinkelman:** Methodology; writing‐review and editing. **Morgana Mongraw‐Chaffin:** Methodology; writing‐review and editing. **John W. Sanders:** Methodology; writing‐review and editing. **Sharon Saydah:** Conceptualization; methodology; writing‐review and editing. **Ian D. Plumb:** Conceptualization; methodology; writing‐original draft; writing‐review and editing.

### PEER REVIEW

The peer review history for this article is available at https://publons.com/publon/10.1111/irv.13080.

## Supporting information


**Table S1:** Associations between COVID‐19 and type of known COVID‐19 contact during the 10 days before the index date, among non‐healthcare workers, November 2020–October 2021
**Table S2:** Differences in characteristics of study participants by whether ≥2 serology results were available
**Table S3:** Gap days between recent survey entry among participants reporting a new positive virologic test
**Table S4:** Comparison of x to Y: Sensitivity analysis limiting to case‐participants with subsequent positive serology
**Figure S1:** New symptoms before self‐reporting positive viral test
**Figure S2:** Difference in mask wearing stratified by reporting a known exposure, among cases
**Box S1:** Daily Questionnaire for Adult participants
**Appendix S1:** Authorship Appendix

## Data Availability

The datasets used and/or analyzed during the current study are available from the corresponding author on reasonable request.
