# Supplementary material for: Association between COVID‐19 and consistent mask wearing during contact with others outside the household—A nested case–control analysis, November 2020–October 2021
Source: Influenza Other Respir Viruses. 2023 Jan 5;17(1):e13080. doi: 10.1111/irv.13080 (PMC9835433; doi:10.1111/irv.13080)
Supplement: Supplementary file 1 — Table S1: Associations between COVID‐19 and type of known COVID‐19 contact during the 10 days before the index date, among non‐healthcare workers, November 2020–October 2021 Table S2: Differences in characteristics of study participants by whether ≥2 serology results were available Table S3: Gap days between recent survey entry among participants reporting a new positive virologic test Table S4: Comparison of x to Y: Sensitivity analysis limiting to case‐participants with subsequent positive serology Figure S1: New symptoms before self‐reporting positive viral test Figure S2: Difference in mask wearing stratified by reporting a known exposure, among cases Box S1: Daily Questionnaire for Adult participants Appendix S1: Authorship Appendix [file IRV-17-e13080-s001.docx]

**Association between COVID-19 and Consistent Mask Wearing during Contact with Others Outside the Household — a Nested Case Control Analysis, November 2020–October 2021**

Ashley H. Tjaden, Sharon L. Edelstein, Naheed Ahmed, Lydia Calamari, Keerti L. Dantuluri, Michael Gibbs, Amy Hinkelman, Morgana Mongraw-Chaffin, John W. Sanders, Sharon Saydah, Ian D. Plumb, and the COVID-19 Community Research Partnership Study Group

**Supplemental Materials**

1. Supplementary Table 1: Associations between COVID-19 and type of known COVID-19 contact during the 10 days before the index date, among non-healthcare workers, November 2020–October 2021
2. Supplementary Table 2: Differences in characteristics of study participants by whether ≥2 serology results were available
3. Supplementary Table 3: Gap days between recent survey entry among participants reporting a new positive virologic test
4. Supplementary Table 4: Sensitivity analysis limiting to case-participants with subsequent positive serology
5. Supplementary Figure 1: New symptoms before self-reporting positive viral test
6. Supplementary Figure 2: Difference in mask wearing stratified by reporting a known exposure, among cases
7. Supplementary Box 1: Daily Questionnaire for Adult participants
8. Authorship Appendix

**Supplemental Tables**

Supplementary Table 1: Associations between COVID-19 and type of known COVID-19 contact during the 10 days before the index date, among non-healthcare workers, November 2020–October 2021

|  | Case-participants ^a^  (N = 107) | Control-participants ^b^  (N = 69) | Unadjusted OR ^c^  (95% CI) | Adjusted OR ^d^  (95% CI) |
| --- | --- | --- | --- | --- |
| If close contact, at least one contact of ≥15 minutes duration^e^ |  |  |  |  |
| No | 18 (16.8%) | 29 (42.0%) | REF | REF |
| Yes | 89 (83.2%) | 40 (58.0%) | **3.58 (1.80, 7.30)** | **3.05 (1.44, 6.59)** |
| If ≥1 contact, of ≤6 feet proximity^f^ |  |  |  |  |
| No | 4 (3.7%) | 16 (23.2%) | REF | REF |
| Yes | 103 (96.3%) | 53 (76.8%) | **7.77 (2.70, 28.19)** | **9.58 (2.85, 40.90)** |
| If ≥1 contact, outside of workplace^g^ |  |  |  |  |
| No | 14 (13.1%) | 21 (30.4%) | REF | REF |
| Yes | 93 (86.9%) | 48 (69.6%) | **2.91 (1.37, 6.33)** | **2.72 (1.09, 6.95)** |

^a^ Self-reported a positive viral test (indicating a positive SARS-CoV-2 antigen or nucleic acid amplification test result) with new COVID-19–like symptoms reported among non-healthcare workers with a known exposure

^b^ No evidence of SARS-CoV-2 infection among non-healthcare workers with a known exposure.

^c^ Unconditional logistic regression among non-healthcare worker participants who reported at least one close contact/known exposure in the 10 days preceding their index date. Due to small sample sizes, these models were not adjusted enrollment site.

^d^ Unconditional logistic regression adjusted for age group, sex, race/ethnicity, county classification, time of match (by 3-month quarters) and vaccination status.

^e^ Participant reported at least one close contact in which they were in contact with the patient (exposure duration) for more than 15 minutes.

^f^ Participant reported at least one close contact in which they were in direct contact or contact less than 6 feet.

^g^ Participant reported at least one close contact in which they were in contact with the patient outside their workplace (e.g. Home, Social Activity or Other)

Supplementary Table 2: Differences in characteristics of study participants by whether ≥2 serology results were available

|  | <2 Serology test results | ≥2 Serology test results | p-value^a^ |
| --- | --- | --- | --- |
| N | 7,398 | 15,697 |  |
| Age in years^b^ |  |  | *<*0.001 |
| 18–44 | 3,329(45.0%) | 4,795(30.5%) |  |
| 45–64 | 2,399(32.4%) | 5,548(35.3%) |  |
| ≥65 | 1,670(22.6%) | 5,354(34.1%) |  |
| Sex^b^ |  |  | 0.012 |
| Female | 4,865(65.8%) | 10,056(64.1%) |  |
| Male | 2,533(34.2%) | 5,641(35.9%) |  |
| Race and ethnicity^b^ |  |  | *<*0.001 |
| Hispanic | 311(4.2%) | 578(3.7%) |  |
| non-Hispanic, Black | 846(11.4%) | 1,142(7.3%) |  |
| non-Hispanic, White | 5,673(76.7%) | 13,125(83.6%) |  |
| Other^c^ | 568(7.7%) | 852(5.4%) |  |
| County of residence |  |  | *<*0.001 |
| Rural | 1,591(21.5%) | 3,625(23.1%) |  |
| Suburban | 1,806(24.4%) | 4,241(27.0%) |  |
| Urban | 4,001(54.1%) | 7,830(49.9%) |  |
| Healthcare worker^b^ |  |  | *<*0.001 |
| No | 5,878(79.5%) | 12,762(81.3%) |  |
| Yes | 1,520(20.5%) | 2,935(18.7%) |  |
| Health system region |  |  | *<*0.001 |
| Deep South | 139(1.9%) | 181(1.2%) |  |
| Mid-Atlantic | 3,551(48.0%) | 8,115(51.7%) |  |
| South East | 3,708(50.1%) | 7,401(47.1%) |  |

^a^P-values from Pearson’s Chi-squared tests for categorical variables and Welch’s two sample t-test for continuous variables.

^b^Self-reported at enrollment

^c^ Other race/ethnicity includes American Indian or Alaskan Native, Asian or Pacific Islander, mixed race/ethnicity, and participants who chose not to specify their race/ethnicity

Supplementary Table 3: Gap days between recent survey entry among participants reporting a new positive virologic test

| **Gap days since most recent survey entry^a^** | **No. participants (%)** |
| --- | --- |
| 0 | 282 (78.6%) |
| 1 | 44 (12.2%) |
| 2 | 16 (4.4%) |
| 3 | 6 (1.7%) |
| 4 | 2 (0.6%) |
| 5 | 3 (0.8%) |
| 6 | 3 (0.8%) |
| 7 | 2 (0.6%) |
| 8 | 1 (0.2%) |

^a^Number of gap days between date of self-reporting positive test and most recent preceding survey.

Participants were only asked about whether was a new positive SARS-CoV-2 test virologic test result since the last survey entry, were not directly asked for the date of the test. The number of days with a gap between the most recent surveys therefore indicates the level of uncertainty in using the response date as a proxy for the test date. The test date could be inferred within approximately 2 days for 342/359 (95%) participants.

Supplementary Table 4: Sensitivity analysis limited to case-participants with subsequent positive serology

|  | Case-participants  (N = 205)^a^ | Control-participants  (N = 2050)^b^ | Unadjusted cOR^c^  (95% CI) | Adjusted cOR^d^  (95% CI) |
| --- | --- | --- | --- | --- |
| Contact with others outside household without a mask |  |  |  |  |
| No | 90 (43.9%) | 1,149 (56.0%) | REF | REF |
| Yes | 115 (56.1%) | 901 (44.0%) | **1.96**  **(1.40, 2.76)** | **1.86**  **(1.29, 2.70)** |
| Contact with others outside household without a mask *by vaccination status* ^f^ |  |  |  |  |
| Unvaccinated | 126 (67.7%) | 939 (52.4%) | **1.90 (1.26, 2.83)** | **1.87 (1.21, 2.86)** |
| ≥14 days after mRNA dose 2 | 60 (32.3%) | 852 (47.6%) | **2.39 (1.15, 5.68)** | **2.62 (1.21, 6.50)** |
| Close contact with person with COVID-19^e^ |  |  |  |  |
| No | 126 (61.5%) | 1,967 (96.0%) | REF | REF |
| Yes | 79 (38.5%) | 83 (4.0%) | **15.34**  **(10.45, 22.51)** | **17.57**  **(10.76, 28.69)** |

^a^ Self-reported a positive viral test (indicating a positive SARS-CoV-2 antigen or nucleic acid amplification test result) with new COVID-19–like symptoms reported among non-healthcare workers with a known exposure

^b^ Control-participants had no serologic or virologic evidence of SARS-CoV-2 infection and were matched to case-participants by survey date.

^c^ Odds ratio calculated by conditional logistic regression

^d^ Odds ratio calculated by conditional logistic regression, adjusted for age group, sex, race/ethnicity, county classification, healthcare worker occupation, and vaccination status (except where used for stratification)

^e^ Reporting close contact with a person with COVID-19 in the ten days preceding the index date.

^f^ Odds ratio for not consistently wearing a mask during contact with others outside the household, compared with consistently wearing a mask, stratified by vaccination status on the index date. Unconditional logistic regression models were adjusted for month of index date. The interaction term for differences by vaccination status was non-significant.

Supplementary Figure 1: New symptoms before self-reporting positive viral test


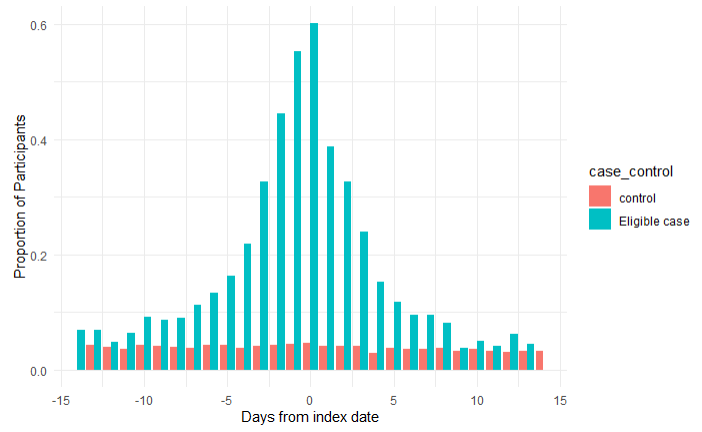


For this figure, the analysis dataset included eligible case-participants before exclusion for having a symptomatic case (N=545), and control-participants (N=3,544). The bar chart shows the proportion of participants reporting at least one “new” symptom (a symptom not reported in the preceding 7 days) for eligible case-participants and date of match for control-participants.

Supplementary Figure 2: Difference in mask wearing stratified by reporting a known exposure, among cases


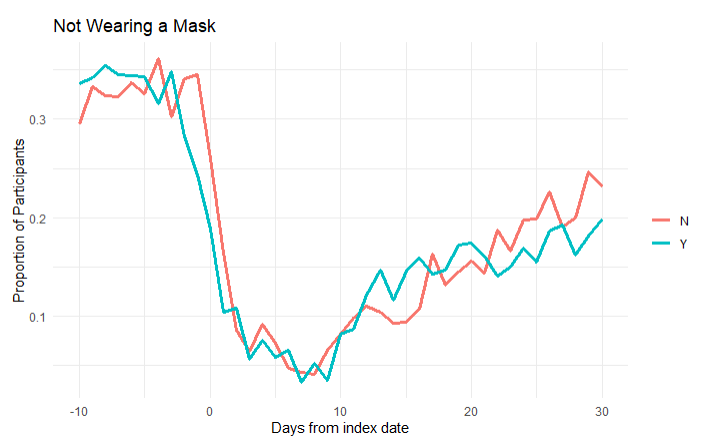


**Supplementary Box 1: Daily Questionnaire for Adult participants**

| Please answer the following questions based on what you are experiencing between your last update and now.   - Overall, do you consider yourself healthy?   - Yes   - No - In the last 24 hours, what symptoms did you experience?   - None   - Fatigue   - Muscle or body aches   - Fever   - Chills   - Congestion or runny nose   - Sore throat   - Cough   - Nausea or vomiting   - Diarrhea   - Headache   - New loss of taste or smell   - New confusion or inability to wake or stay awake   - Bluish lips or Face   - Persistent pain or pressure in the chest   - Shortness of breath or difficulty breathing - If YES to anything above:   - Have you sought treatment for symptoms? - If YES,   - Were you hospitalized? - If YES to fever:   - Highest temperature in last 24 hours - In the last 24 hours, have you worn a face mask or face covering every time you interacted with others (not in your household) within a distance of less than 6 feet? - Wore a mask - Did not wear a mask - No interactions - Did you have close contact with someone who has tested positive for COVID-19 infection? - Yes - No - I don’t know - If YES contact:   - When was your most recent close contact with this person?     - Last 24 hours     - Within the last 7 days     - Within 1-2 weeks ago     - More than 2 weeks ago   - Where did you come in contact with this patient?     - Home     - Work     - Social activity     - Other   - How long were you in contact with the patient?     - Less than 1 minute     - 1 to 15 minutes     - More than 15 minutes   - How close was your contact?     - Direct contact     - Less than 6 feet     - More than 6 feet - Do you have any new test results for COVID-19?   - Yes   - No   - Type of test     - COVID-19 infection (nasal swab, saliva or spit)     - COVID-19 antibody test (blood)     - Neither/I don’t know   - What were the test results?     - Positive     - Negative     - Unknown - Have you received a vaccine for COVID-19, since the last survey?   - Yes   - No   - If YES vaccine:     - Was this part of a clinical trial?       - Yes       - No       - I don’t know     - Which vaccine:       - I don’t know       - Moderna – MRNA       - Pfizer – BNT162       - Other     - Vaccination date? (If Known) |
| --- |

**Authorship Appendix: The COVID-19 Research Group** (*Site Principal Investigator)

**Wake Forest School of Medicine:** Thomas F Wierzba PhD, MPH, MS*, John Walton Sanders, MD, MPH, David Herrington, MD, MHS, Mark A. Espeland, PhD, MA, John Williamson, PharmD, Morgana Mongraw-Chaffin, PhD, MPH, Alain Bertoni, MD, MPH, Martha A. Alexander-Miller, PhD, Paola Castri, MD, PhD, Allison Mathews, PhD, MA, Iqra Munawar, MS, Austin Lyles Seals, MS, Brian Ostasiewski, Christine Ann Pittman Ballard, MPH, Metin Gurcan, PhD, MS, Alexander Ivanov, MD, Giselle Melendez Zapata, MD, Marlena Westcott, PhD, Karen Blinson, Laura Blinson, Mark Mistysyn, Donna Davis, Lynda Doomy, Perrin Henderson, MS, Alicia Jessup, Kimberly Lane, Beverly Levine, PhD, Jessica McCanless, MS, Sharon McDaniel, Kathryn Melius, MS, Christine O’Neill, Angelina Pack, RN, Ritu Rathee, RN, Scott Rushing, Jennifer Sheets, Sandra Soots, RN, Michele Wall, Samantha Wheeler, John White, Lisa Wilkerson, Rebekah Wilson, Kenneth Wilson, Deb Burcombe, Georgia Saylor, Megan Lunn, Karina Ordonez, Ashley O’Steen, MS, Leigh Wagner.

**Atrium Health:** Michael S. Runyon MD, MPH*, Lewis H. McCurdy MD*, Michael A. Gibbs, MD, Yhenneko J. Taylor, PhD, Lydia Calamari, MD, Hazel Tapp, PhD, Amina Ahmed, MD, Michael Brennan, DDS, Lindsay Munn, PhD RN, Keerti L. Dantuluri, MD, Timothy Hetherington, MS, Lauren C. Lu, Connell Dunn, Melanie Hogg, MS, CCRA, Andrea Price, Marina Leonidas, Melinda Manning, Whitney Rossman, MS, Frank X. Gohs, MS, Anna Harris, MPH, Jennifer S. Priem, PhD, MA, Pilar Tochiki, Nicole Wellinsky, Crystal Silva, Tom Ludden PhD, Jackeline Hernandez, MD, Kennisha Spencer, Laura McAlister.

**MedStar Health Research Institute:** William Weintraub MD*, Kristen Miller, DrPH, CPPS*, Chris Washington, Allison Moses, Sarahfaye Dolman, Julissa Zelaya-Portillo, John Erkus, Joseph Blumenthal, Ronald E. Romero Barrientos, Sonita Bennett, Shrenik Shah, Shrey Mathur, Christian Boxley, Paul Kolm, PhD, Ella Franklin, Naheed Ahmed, Moira Larsen.

**Tulane**: Richard Oberhelman MD*, Joseph Keating PhD*, Patricia Kissinger, PhD, John Schieffelin, MD, Joshua Yukich, PhD, Andrew Beron, MPH, Johanna Teigen, MPH.

**University of Maryland School of Medicine:** Karen Kotloff MD*, Wilbur H. Chen MD, MS*, DeAnna Friedman-Klabanoff, MD, Andrea A. Berry, MD, Helen Powell, PhD, Lynnee Roane, MS, RN, Reva Datar, MPH, Colleen Reilly.

**University of Mississippi**: Adolfo Correa MD, PhD*, Bhagyashri Navalkele, MD, Yuan-I Min, PhD, Alexandra Castillo, MPH, Lori Ward, PhD, MS, Robert P. Santos, MD, MSCS, Pramod Anugu, Yan Gao, MPH, Jason Green, Ramona Sandlin, RHIA, Donald Moore, MS, Lemichal Drake, Dorothy Horton, RN, Kendra L. Johnson, MPH, Michael Stover.

**Wake Med Health and Hospitals:** William H. Lagarde MD*, LaMonica Daniel, BSCR.

**New Hanover:** Patrick D. Maguire MD*, Charin L. Hanlon, MD, Lynette McFayden, MSN, CCRP, Isaura Rigo, MD, Kelli Hines, BS, Lindsay Smith, BA, Monique Harris, CCRP, Belinda Lissor, AAS, CCRP, Vivian Cook, MA, MPH, Maddy Eversole, BS, Terry Herrin, BS, Dennis Murphy, RN, Lauren Kinney, BS, Polly Diehl, MS, RHIA, Nicholas Abromitis, BS, Tina St. Pierre, BS, Bill Heckman, Denise Evans, Julian March, BA, Ben Whitlock, CPA, MSA, Wendy Moore, BS, AAS, Sarah Arthur, MSW, LCSW, Joseph Conway.

**Vidant Health:** Thomas R. Gallaher MD*, Mathew Johanson, MHA, CHFP, Sawyer Brown, MHA, Tina Dixon, MPA, Martha Reavis, Shakira Henderson, PhD, DNP, MS, MPH, Michael Zimmer, PhD, Danielle Oliver, Kasheta Jackson, DNP, RN, Monica Menon, MHA, Brandon Bishop, MHA, Rachel Roeth, MHA.

**Campbell University School of Osteopathic Medicine**: Robin King-Thiele DO*, Terri S. Hamrick PhD*, Abdalla Ihmeidan, MHA, Amy Hinkelman, PhD, Chika Okafor, MD (Cape Fear Valley Medical Center), Regina B. Bray Brown, MD, Amber Brewster, MD, Danius Bouyi, DO, Katrina Lamont, MD, Kazumi Yoshinaga, DO, (Harnett Health System), Poornima Vinod, MD, A. Suman Peela, MD, Giera Denbel, MD, Jason Lo, MD, Mariam Mayet-Khan, DO, Akash Mittal, DO, Reena Motwani, MD, Mohamed Raafat, MD (Southeastern Health System), Evan Schultz, DO, Aderson Joseph, MD, Aalok Parkeh, DO, Dhara Patel, MD, Babar Afridi, DO (Cumberland County Hospital System, Cape Fear Valley).

**George Washington University Data Coordinating Center:** Diane Uschner PhD*, Sharon L. Edelstein, ScM, Michele Santacatterina, PhD, Greg Strylewicz, PhD, Brian Burke, MS, Mihili Gunaratne, MPH, Meghan Turney, MA, Shirley Qin Zhou, MS, Ashley H Tjaden, MPH, Lida Fette, MS, Asare Buahin, Matthew Bott, Sophia Graziani, Ashvi Soni, MS, Guoqing Diao, PhD, Jone Renteria, MS.

**George Washington University Mores Lab:** Christopher Mores, PhD, Abigail Porzucek, MS.

**Oracle Corporation:** Rebecca Laborde, Pranav Acharya.

**Sneez LLC**: Lucy Guill, MBA, Danielle Lamphier, MBA, Anna Schaefer, MSM, William M. Satterwhite, JD, MD.

**Vysnova Partners:** Anne McKeague, PhD, Johnathan Ward, MS, Diana P. Naranjo, MA, Nana Darko, MPH, Kimberly Castellon, BS, Ryan Brink, MSCM, Haris Shehzad, MS, Derek Kuprianov, Douglas McGlasson, MBA, Devin Hayes, BS, Sierra Edwards, MS, Stephane Daphnis, MBA, Britnee Todd, BS.

**Javara Inc:** Atira Goodwin.

**External Advisory Council:** Ruth Berkelman, MD, Emory, Kimberly Hanson, MD, U of Utah, Scott Zeger, PhD, Johns Hopkins, Cavan Reilly, PhD, U. of Minnesota, Kathy Edwards, MD, Vanderbilt, Helene Gayle, MD MPH, Chicago Community Trust, Stephen Redd.
